# Mixed Signals in Child and Adolescent Mental Health and Well‐Being Indicators in the United States: A Call for Improvements to Population Health Monitoring

**DOI:** 10.1111/1468-0009.12634

**Published:** 2023-04-13

**Authors:** NATHANIEL W. ANDERSON, NEAL HALFON, DANIEL EISENBERG, ANNA J. MARKOWITZ, KRISTIN ANDERSON MOORE, FREDERICK J. ZIMMERMAN

**Affiliations:** ^1^ University of California Los Angeles Jonathan and Karin Fielding School of Public; ^2^ University of California Los Angeles David Geffen School of Medicine; ^3^ University of California Los Angeles Meyer and Renee Luskin School of Public Affairs; ^4^ University of California Los Angeles Graduate School of Education and Information Studies; ^5^ Child Trends

**Keywords:** population health, mental health, child health, adolescent health, well‐being, population surveillance, health status indicators

## Abstract

**Policy Points**

- Social indicators of young peoples’ conditions and circumstances, such as high school graduation, food insecurity, and smoking, are improving even as subjective indicators of mental health and well‐being have been worsening. This divergence suggests policies targeting the social indicators may not have improved overall mental health and well‐being.
- There are several plausible reasons for this seeming contradiction. Available data suggest the culpability of one or several common exposures poorly captured by existing social indicators.
- Resolving this disconnect requires significant investments in population‐level data systems to support a more holistic, child‐centric, and up‐to‐date understanding of young people's lives.

Monitoring population health is one of the 10 essential services of public health.^1^ Accurate information on current and emerging threats to the public's health forms the basis for high‐quality research, practice, and policy. For this reason, critical appraisal of the measures and data systems tracking the nation's health is a persistent necessity.

A growing body of research highlights the importance of childhood mental health and well‐being over the rest of the lifecourse.^2^, ^3^, ^4^ Currently, we use two data streams to examine the extent to which children and adolescents are thriving. The first data stream includes indicators of the specific conditions and circumstances of young people, such as rates of low birthweight, obesity, food insecurity, reading and math proficiency, high school graduation, substance use, and similar factors. In what follows, we will refer to such measures as *social indicators*, because they are the metrics by which the population benefits of social policy are commonly assessed. The second data stream includes direct measures of mental health and well‐being, such as symptoms of depression and anxiety as well as measures of positive mental and emotional health such as life satisfaction and self‐esteem. In what follows, we will refer to such measures as *subjective indicators of mental health*, because they are based on self‐reported perceptions, feelings, and experiences.

This essay assesses the available information on population‐level mental health and well‐being for young people in the United States and explores candidate reasons why the social indicators are moving in the opposite direction from the subjective indicators. Because of the limitations of existing data, we cannot determine for certain what is causing this divergence. Instead, we provide recommendations for how health monitoring can be strengthened to better understand what matters to young people, and design appropriate supports for their thriving.

## Measures of Child and Adolescent Thriving

Together, the social and the subjective indicators provide complementary views of child and adolescent thriving. While distinct conceptually, they might be expected to move in tandem. Children and adolescents who graduate high school on time and never experience food insecurity can be expected to have higher self‐esteem and lower anxiety relative to their disadvantaged counterparts, since educational attainment and financial security in adulthood are consistently correlated with better mental health.^5^, ^6^, ^7^, ^8^, ^9^, ^10^, ^11^ Hence at the population level, times and places that have high values for social indicators should similarly be expected to have greater levels of subjectively experienced mental health and well‐being. Although the correlation should not be expected to be perfect,^12^ it should be observable; and, indeed, for several decades there hadbeen a close relationship between these indicators. For example, analysis of the Child and Youth Well‐Being Index (CWI),an aggregation of national social indicatorsfrom 1975 to 2003 documented a strong correlation (ρ = 0.65) with a measure of life satisfaction from a nationally representative sample of high schoolers from the Monitoring the Future study.^13^

One of the benefits of using social indicators to measure children's mental health and well‐being is that they are collected with high frequency and subgeographic granularity. Population‐level information on high school graduation and birthweight are collected annually, and other measures such as obesity and tobacco use are routinely sampled through instruments like the Youth Risk Behavior Surveillance Survey and the National Survey on Drug Use and Health, each of which has an annual sample size approaching 15,000 youth. The density of this data makes visible the trends in the social indicators over time, across states, and often within states. Such dimensionality helps researchers track emerging issues and make inferential comparisons across jurisdictions with different policy regimes for research, practice, and policymaking purposes.

To further facilitate comparisons, social indicators are often aggregated into a single composite index of thriving. Severalexamples includethe aforementioned CWI, and the more recent and widely used Annie E. Casey Foundation's KIDS COUNT Index.^14^, ^15^

In contrast to the data on social indicators, there are several well‐documented weaknesses in population health monitoring systems for existing subjective mental health and well‐being indicators.^16^, ^17^ First, the few measures that are collected at scale often face critical limitations, such as comparability over time, reliance on parent‐report, and single point‐in‐time measurement. Second, the sparseness of data across geography, race/ethnicity, and class restricts the set of statistical tools available for hypothesis testing, and inhibits necessary efforts towards documenting inequities. Third, monitoring is spread over several different data systems, each with their own particular focus. This raises challenges for comprehensive analysis of mental health's various social, political, commercial, and other nonmedical determinants.

**Figure 1:**
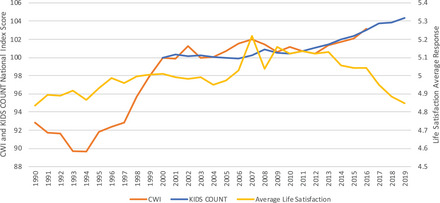
National Trends in Social and Subjective Indiciators of Child and Adolescent Mental Health and Well‐Being in the United States, 1990‐2019. [Colour figure can be viewed at wileyonlinelibrary.com] Source: Child and Youth Well‐Being Index (CWI) is available from Ken Land and coauthors at https://www.soc.duke.edu/∼cwi/. Annie E. Casey KIDS COUNT Index is reconstructed by the authors. Underlying data is available from https://datacenter.kidscount.org/data#USA/1/0/char/0. Life Satisfaction is from the Monitoring the Future Study and available from https://www.icpsr.umich.edu/web/NAHDAP/studies/30985#. Notes: KIDS CWI and KIDS Count estimates are linearly rescaled such that they are 100 in the year 2000. The units represent the percent change relative to that base year. Life satisfaction is from a global measure asked among a nationally representative sample of 12th graders each year based on the following instrument: “How satisfied are you with your life‐as‐a‐whole these days?” The answer range is a seven‐point Likert rating scale: Completely Dissatisfied, Quite Dissatisfied, Somewhat Dissatisfied, Neither Satisfied or Dissatisfied, Somewhat Satisfied, Quite Satisfied, and Completely Satisfied. ^14^, ^18^, ^19^

These weaknesses of existing subjective mental health and well‐being indicators, alongside the greater density and consistency of the social indicators, has resulted in a public health monitoring that skews substantially towards the social indicators. As a result, beyond being tracked for their own value, the social indicators have taken on a secondary role of proxying for the subjective indicators. However, this was only defensible because for decades the social indicators performed reasonably well in this function.^13^ Since 2012 or so, this has changed: social indicators have continued to improve, while subjective indicators have sharply declined. Figure 1 presents this pattern visually, tracking trends in two social indicator composite indices, the CWI and KIDS COUNT index, alongside an average life satisfaction score using a seven‐point scalar instrument from the Monitoring the Future Study from 1990 through 2019.^14^, ^18^, ^19^

In addition to the downturn in average life satisfaction among adolescents, similar trends over the same period have been found for a distinct but related subcategory of subjective indicators, which we refer to as *mental distress*. These are a set of nonspecific, internalized symptoms ranging in severity from subclinical levels that can still greatly affect general quality of life, to more acute levels potentially resulting in self‐harm or suicide. ^20^, ^21^, ^22^ The declines in measures of mental distressare further consistent with clinical studies documenting rising levels of mental health–related hospitalizations and expenditures, and higher rates of suicide‐related outcomes.^23^, ^24^

This divergence between the social and the subjective indicators of mental health and well‐being is deeply concerning. Mixed signals from systems of mental health monitoring make it difficult to identify a policy response that is truly in the best interests of children and adolescents. When overall progress on the social indicators no longer translates into improvements in subjective mental health and well‐being, it suggests that policymakers cannot rely only on these indicators—or their composite indices—to inform their actions.

## Why are Subjective Indicators Diverging from Social Indicators?

Having established the divergence between the social and subjective indicators of mental health and well‐being, we now ask why is this occurring? Below we explore several possibilities.

### Are Summary Measures of Trends in the Social Indicators Reliable?

**Figure 2:**
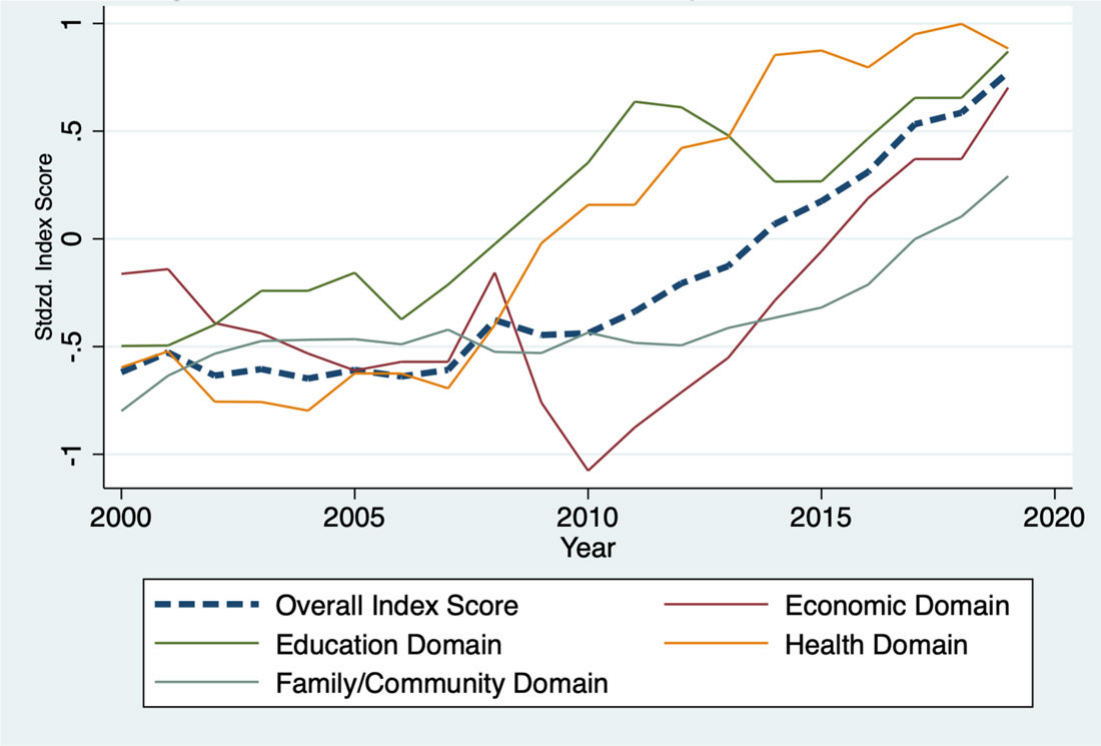
Authors’ Reconstruction of the Annie E. Casey KIDS COUNT Index, 2000‐2019. [Colour figure can be viewed at wileyonlinelibrary.com] Source: The Annie E. Casey Foundation. 2021 Kids Count Data Book: State Trends in Child Wellbeing. Baltimore, MD2021. Notes: Authors’ reconstruction of Annie E. Casey KIDS COUNT Index. Overall and domain index scores are standardized across all state‐year combinations. While the KIDS COUNT index has changed over time, we apply the most recent methodology to all years of data shown here, based on the redesign which occurred in 2011. This version of the index reverts to using the measure of illicit drug use, instead of obesity, which was changed in the 2020 chartbook.^18^

It is plausible that the trend of overall improvement exhibited by summary indices such as KIDS COUNT provides a false sense of simplicity by obfuscating heterogeneity in trends across social indicators. To examine this possibility, Figure 2 reconstructs the KIDS COUNT index for 2000–2019 with more detail. In addition to the clear upward long‐term trend, subanalysis of the four independent domains of education, economics, health, and family/community reveal that the overall improvement in the index is not domain specific. Rather, each of the domains of well‐being are moving in the same direction.

Furthermore, both the KIDS COUNT and the CWI indices are composed of a specific set of indicators chosen by experts and aggregated with equal weights into an index, but this procedure may be inaccurate, as it does not solicit information from young people directly on the social indicators most important to their subjective experiences. To address this issue, recent work employed a comprehensive‐inventory approach to identifying candidate indicators as well as a data‐driven approach to estimating their respective weights to maximize the predictive power of the resulting index.^25^ However, follow‐up work similarly found that population‐level estimates based on this methodology continued to improve from 2012 to 2019, in parallel with the KIDS COUNT Index.^26^

The consistent findings across social indicators, and the various methodologies for aggregating them, undermine some of the hypothesized explanations for their divergence with the subjective indicators. Examples of such hypotheses include lower economic opportunity in the wake of the Great Recession^27^, ^28^ and rising adversity in family contexts such as higher rates of parental incarceration and opioid misuse.^29^, ^30^, ^31^, ^32^, ^33^ Although these theories may contribute to the declines in subjective indicators, they are not satisfying *on their own* as explanations for the divergence with the social indicators for several reasons. First, we would expect these substantial changes in material circumstances to be at least somewhat accounted for by the social indicators. Furthermore, the similar decline in subjective indicators by race and socioeconomic status suggests that these theories are at best secondary or downstream from the forces that are negatively impacting mental health because these hypotheses would be more likely to manifest in greater effects for disadvantaged populations.

### Are the Mechanisms of Mental Health and Well‐Being Changing Over Time?

Another explanation for the weakening relationship between the social and subjective indicators is that it may simply be a natural occurrence over the long run as a result of societal progress. For young people in the United States, increases in high school graduation and reductions in cigarette smoking represent genuine policy successes.^34^, ^35^, ^36^ However, the growth in compensation for those with a high school degree has not kept pace with their college peers,^37^ and adolescents engaging in substance use have been shifting to other modalities such as vaping.^38^ To be clear, these illustrations do not imply that policy should not continue to address these social indicators—as we have already mentioned, they are important to the current and future health of young people.^39^, ^40^, ^41^ Rather, these examples suggest that population indices which prioritize consistency in their component measures may naturally lose predictive power of the subjective indicators over time. As such, the existing sets of social indicators may require periodic updating with additional complementary measures.

### Are Existing Social Indicators Overly Deficit and Future Oriented?

One of the strongest critiques of how society measures the health and well‐being of young people is that it reinforces a perception of children and adolescent as incomplete or unrealized adults.^42^ Put more simply, historically the emphasis has been on children's *well‐becoming* rather than their present *well‐being*.^43^ Although well‐intentioned, this somewhat paternalistic focus has led to public policy undervaluing assets‐based and present‐oriented aspects of a healthy and happy childhood. Instead, measurement of health and social issues has focused on deficits, such as obesity or food insecurity, and on future‐oriented outcomes such as high school dropout.^44^ This absence of certain facets of well‐being within the social indicators could contribute to their divergence over time with the subjective indicators if there is a newly emerging threat to in‐the‐moment experiences and sense of self, but not future outcomes such as high school graduation.

### Are There Distributional Effects?

The outcome indicators of well‐being are dichotomous measures that may not be sensitive to what is occurring within certain segments of the population. One clear example of this has been the recent declines in subjective well‐being observed among more privileged young persons,^21^, ^22^ who are still highly unlikely to drop out of high school or experience other forms of material hardship. Another illustration of this can be seen when looking at the outcome distribution of poor‐mental‐health‐days over time among young adults from the Behavioral Risk Factor Surveillance System (Appendix Figure 1). Respondents towards the bottom portion of the outcome distribution (10^th^ and 25^th^ percentiles) reported 5 additional days of poor mental health in 2019 compared to 2012, whereas the corresponding increase for the median respondent was 2 additional days. In this case, a dichotomized measure, such as "having at least one day in poor mental health in the past month", may understate what is happening more generally within the broader population, because it treats someone with 10 poor mental health days and 20 poor mental health days equally.

### Have There Been Changes in Diagnoses Rates and Willingness to Report over Time?

Some of the increase in mental‐illness reporting may be driven by advances in insurance coverage and access to mental health care,^20^ as well as greater levels of mental health literacy and social acceptability. However, the sudden and dramatic changes beginning in 2012 make this explanation implausible. At best, it can only partially explain some of the magnitude of the divergence,^21^ but does not address why the trends are moving in different directions. Furthermore, this potential explanation is seriously weakened by the parallel increase in suicide among youth over the same period, suggesting there has been a real and substantive increase in mental distress.^24^

### Is Social Media Use a Direct Biopsychosocial Cause of Declining Mental Health?

The suddenness of the decline in subjective indicators of mental health and well‐being, the consistency in the decline by race/ethnicity and socioeconomic status, and the greater magnitude among girls, has led some to postulate the widespread adoption of social media as the underlying cause for these phenomena.^21^, ^22^, ^45^ Potential mechanisms include reductions in sleep quality,^46^ increased exposure to cyberbullying,^47^ and greater salience of unhealthy social comparisons.^48^

However, studies of social media use and the subjective indicators among individuals have to this point identified relatively small associations and typically lacked rigorous causal study design.^49^, ^50^ Yet, especially here, an absence of evidence does not imply evidence of absence. In particular, these studies do not rule out population‐level effects, for the reason identified by Geoffrey Rose in his seminal work “Sick Individuals and Sick Populations”:^51^ they may be looking at small variations in use amongst teens, whereas nearly *all* teenagers are using these forms of communication far more than previously. Additionally, the typical metrics for social media use, such as the duration of daily exposure, capture a narrow set of mechanisms related to its theorized negative impact. Specific and easily quantifiable aspects, such as the types of websites/platforms users interact with and the behaviors that characterize their engagement, remain largely understudied despite acknowledgement of its potentially critical importance.^52^, ^53^

### Is Social Media an Indirect Cause Affecting Young People's Optimism About the Future?

Another candidate explanation for the divergence in social and subjective indicators is that despite the improvements in present‐day conditions and circumstances, many youth may be feeling less optimistic about the future. Though we argued that recent challenges to well‐being, including the Great Recession, Mass Incarceration, and the Opioid Epidemic, are unlikely to be direct causes of the divergence in their own right, it is also clear that young people are increasingly concerned about their opportunity to thrive in a world with such challenges.^54^, ^55^, ^56^ Other macro–“21^st^‐century challenges,” such as climate change, political radicalization, resistance towards efforts for racial justice, and COVID‐19, have emerged as central concerns to young people,^57^, ^58^, ^59^ and have been linked to their well‐being in other research.^60^, ^61^, ^62^, ^63^, ^64^, ^65^, ^66^, ^67^

It is unclear to what extent such emerging challenges may be a cause for the sharp decrease in mental health in their own right. What is clear is that social media has been used—sometimes deliberately^68^, ^69^—to exacerbate outrage and fear about these issues. Such amplification of the tension and anxiety around these issues may be broadly harmful to young people. As such, instead of acting primarily as a biopsychosocial cause, social media may operate more indirectly as a *cultural aggravator* of the stress of other 21^st^‐century challenges.

What makes both of these social‐media‐focused hypotheses plausible explanations for the divergence between the social and subjective indicators is that the current social indicators are poorly designed to capture their broader effects. Metrics like social media usage are largely nonexistent in population‐level monitoring, and there is little evidence to suggest they affect existing social indicators like test scores or high school graduation.^49^

### Is the Divergence a Period Effect or a Cohort Effect?

In the absence of additional measures or more integrated data sources to enable more sophisticated semicausal analyses, Age‐Period‐Cohort (APC) methods provide an attractive tool for assessing the possibility of social media as a either a direct biopsychosocial cause or an indirect cultural aggravator of declining subjective indicators of mental health and well‐being among young people.^70^ APC methods evaluate the presence of independent age, period, and cohort linear effects within an overall trend, using a regression framework. By “age,” we mean changes in subjective indicators may be a function of biological development processes; by “period,” we mean changes in subjective indicators may be driven by an event or exposure affecting individuals of all ages at a point in time; by “cohort,” we mean a combination of the two: a time‐specific event or exposure affecting subjective indicators among persons of a specific developmental stage.

Simply regressing a subjective indicator like mental distress against all three effects is not possible because of multicollinearity (period = cohort + age). APC models make additional assumptions about the relationship of age, period, and cohort to solve this problem. The Appendix Material includes more information about the methodology.^71^

Although these models cannot definitively resolve the specific mechanism for a trend, they can provide compelling evidence that the patterns in the subjective indicators are driven by a common exposure equally affecting *all age groups* in a particular period rather than one that affects certain age groups disproportionately. If the mechanism is specific to a narrow age window, then we would expect to see evidence of primarily *cohort effects*. In contrast, we should see stronger evidence of *period effects* if the exposure affects all age groups relatively equally.

**Figure 3:**
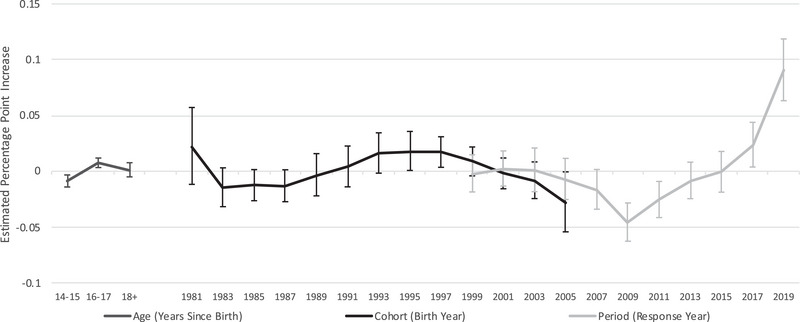
Age‐Period‐Cohort Effects: Sad or Hopeless for 2 Weeks in Past Year, Ages 14‐19. Source: Youth Risk Behavior Survey, 1999‐2019. Notes: Y‐axis shows effect of age, period, or cohort relative to mean for all age‐period‐cohort combinations. X‐axis shows year of effect for period effects, year of birth for cohort effects, and two‐year age group for age effects. Estimates use intrinsic estimator accounting for weights and clustered standard errors (see *apc_ie* downloadable command in STATA). Centers for Disease Control and Prevention. 1999‐2019 Youth Risk Behavior Survey Data. Available at: www.cdc.gov/yrbs. Accessed on February 24, 2022.

Application 1: APC Effects in Mental Distress Among Adolescents (14–19 Years Old). We apply APC methods to data from the Youth Risk Behavior Surveillance System, which asks high schoolers whether they were feeling sad or hopeless for two consecutive weeks in the past year such that they stopped doing regular activities. Figure 3 shows the independent age, period, and cohort effects for a nationally representative repeated cross‐section of high schoolers, where the y‐axis displays the estimated percentage‐point increase in sadness/hopelessness relative to a reference group, the average across all age–period–cohort combinations. As has been found for other measures of mental distress,^21^, ^22^ the results confirm the presence of period effects in recent years: in 2019, adolescents were 9.1 percentage points [95% CI, 6.3–11.9] more likely than the reference group to report sadness/hopelessness. This is 13.7percentage points higher relative to a decade before (β_2009_ = –4.6 [95% CI, –6.3 to –2.8]). By comparison, changes in cohort effects are of a smaller magnitude (maximum, β_1995–1996_ = 1.8 [95% CI, 0.1–3.6]; minimum, β_2005–2006_ = –2.8 percentage points [95% CI, –5.4 to –0.1]).

Application 2: APC Effects in Mental Distress Among Young Adults (18–25 Years Old). As concerning as these findings for adolescents are on their own, they may actually understate the threat of a potential common exposure on broader population health. Given the increased levels of mental distress in adolescence, we could expect several possibilities for trends among young adults (18–25 years old) over the same period:

1. No effects on young adults (i.e., no period or cohort effects): Increases in mental distress are temporary and fade out as adolescents reach adulthood. Any change in distress among adolescents is unique to that period of development or are of secondary importance relative to the stresses of transitioning to adulthood.
2. Effects on young adults emerge at a subsequent date (i.e., cohort effects without period effects): There is no immediate effect on young adults, even for the same period in which adolescents begin experiencing increasing distress. But as these adolescents become adults, their distress persists.
3. Similar trends for young adults (i.e., period effects only): Common exposures affecting adolescents have a similar effect on young adults but without any additional carryover as adolescents age into young adulthood.
4. Cumulative worsening in young adulthood (i.e., period and cohort effects): Common exposures have long‐lasting effects on adolescents *and* independent effects on young adults. Thus, as adolescents age into young adulthood, these population‐level impacts stack on top of one another.

**Figure 4:**
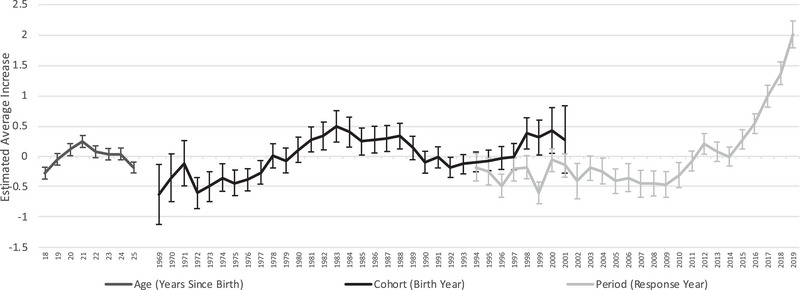
Age‐Period‐Cohort Effects: Poor Mental Health Days, Ages 18‐25. Source: Behavioral Risk Factor Surveillance System (BRFSS). Notes: Y‐axis shows effect of age, period, or cohort relative to mean for all age‐period‐cohort combinations. X‐axis shows year of effect for period effects, year of birth for cohort effects, and age group for age effects. Estimates use intrinsic estimator accounting for weights and clustered standard errors (see *apc_ie* downloadable command in STATA). BRFSS Survey redesign between 2010 and 2011 threatens comparability between the two periods. Centers for Disease Control and Prevention (CDC). Behavioral Risk Factor Surveillance System Survey Data. Atlanta, Georgia: U.S. Department of Health and Human Services, Centers for Disease Control and Prevention, 1994‐2019.

To investigate these possibilities, we applied APC analysis to a measure of poor mental health days in the past month for young adults (18–25 years old) from the Behavioral Risk Factor Surveillance System. Figure 4 shows the results, where the y‐axis represents the average number of additional poor‐mental‐health days for any age, period, or cohort relative to the reference group, again the average for all age‐period‐cohorts combined. The estimates show a clear increase in period effects by 2016 (β_2016_ = 0.54 [95% CI, 0.38–0.70]). By 2019, the period effect is 2.01additional poor mental health days (95% CI, 1.79–2.23) relative to the reference group. There is also evidence of cohort effects for persons born between 1998 and 2000, although these estimates are less precise (β_1998_ = 0.38 [95% CI, 0.12–0.64]; β_2000_ = 0.43[95% CI, 0.05–0.80]). Appendix Figures 2–7 show that the effects are relatively consistent across sex, income, and educational attainment.

We also conduct a similar analysis for adults aged 26–35 years old (Appendix Figure 8). While we note a similar spike in poor mental health days in recent years, the magnitude is smaller to those of the period effects for the population aged 18–25 years old (β_2016_ = 0.19[95% CI, 0.06–0.32]; β_2019_ = 1.13 [95% CI, 0.97–1.28]).

Key Takeaways From APC Analysis. To summarize, we observe large and increasing period effects in subjective indicators of mental distress among adolescents and young adults, the latter of whom additionally experienced cohort effects. By contrast, the accumulation of these effects is smaller for adults aged 26–35 years old, who by and large had completed high school prior to the increases in mental distress among adolescents. Altogether, these results lend support to the idea that a sustained common exposure is responsible for the increasing levels of mental distress among adolescents and young adults during the 2010s. Furthermore, the absence of cohort effects among adolescent individuals casts doubt on explanations that develop slowly and generationally, such as increased vulnerability resulting from overprotective practices by parents and institutions.^72^ Additionally, for adolescents specifically, there is emerging evidence that the consequences of this exposure have lasted into adulthood.

## Recommendations

The problem is clear: indicators of subjective mental health and well‐being have been declining at the same time the social indicators of circumstances and conditions have been improving. Several societal developments in the past decade have been posited as driving these patterns, but existing population data sources are unable to adjudicate between the many possibilities. As such, we offer the following recommendations to improve the available information and ensure research and policy are well‐positioned to monitor population health in a changing world that is presenting new and different challenges to child and adolescent mental health and well‐being.

*Expand Collection of Subjective Indicators of Mental Health and Well‐Being Data in Population Health Monitoring*. Additional information on the subjective indicators, based on measures collected directly from children, is necessary for a more holistic understanding of young peoples’ mental health. First, subjective well‐being measures should be added to more *nationally‐representative repeated cross‐sectional data sources*. The Monitoring the Future survey already has several, but including these measures on other surveys would improve the ability to assess a broader array of potential determinants of child and adolescent mental health.^73^ Second, additional measures capturing aspects beyond mental distress, happiness, and life satisfaction should be incorporated so as to better reflect the multidimensional nature of mental health and well‐being as it pertains to young people.^74^, ^75^ One area that deserves particular attention is the philosophical construct referred to as *eudaimonia*, which captures the concept of meaning and self‐actualization, through aspects such as self‐discovery, development of mastery, and a sense of purpose. Recent empirical analyses have found that these types of measures complement happiness and life satisfaction when describing positive mental health.^74^, ^76^ Fortunately, several validated instruments specifically designed for children are available.^77^, ^78^, ^79^ Third, high‐density data for regional and subpopulation trends are urgently needed. This can empower local policymakers with richer information about their constituents and allow researchers to more fully investigate how structural conditions like patriarchy and structural racism intersect with public policy to produce inequities in well‐being.^80^ Fourth, designing longitudinal data collection strategies will allow research to determine the developmental inputs and inflection points that may help explain how these conditions emerge over the life course.^2^

*Collect Additional Social Indicators to More Fully Capture Mental Health and Well‐Being*. Our call for greater efforts to collect subjective indicators, and the observation that the current social indicators are no longer able to serve in their secondary role as proxies for the subjective indicators, could be interpreted as a general disdain for the social indicators as a means of assessing population‐level well‐being. This is not the case, as the objective measures found in the social indicators have vital and important strengths,^81^, ^82^ particularly when making comparisons across time and space. We fully agree with the conclusions of other scholars that *both* social and subjective indicators are needed as complements to one another.^83^ Therefore, to make the social indicators more effective, they need to be expanded into new areas so as to better capture major drivers of mental health and well‐being, particularly those that focus on present‐oriented aspects of the respondent's experiences and feelings. Adding measures on social media use; validated measures of relationships with friends, family, and peers;^84^, ^85^ opportunities to play;^86^ exposure to extreme climate events;^87^ material deprivation;^88^ and positive childhood experiences^44^, ^89^ would broaden the available social indicators so that composite summary indices like KIDS COUNT more accurately reflect underlying mental health and well‐being. As a part of this, it will be important to continue ongoing efforts to distinguish individual‐level and contextual‐level social indicators, because the latter should be set aside as mediators that can be used to assess how public policy impacts young people's mental health and well‐being.^25^, ^90^, ^91^

*Build Consensus Around Practical Challenges of Data Collection*. There are substantial challenges to high‐quality data collection, especially around subjective indicators. At what age is it appropriate to solicit this information directly through children via broad‐based surveys? How often should this data be collected so that public policy can be more responsive? Given the costs of such an undertaking, what is the balance at which saturation of information has occurred? How can comparability of data over time be maintained, particularly as innovations in data collection cause health monitoring strategies to continue to evolve? These questions do not have clear and obvious answers, so future work should explore these issues, preferably drawing upon a wide array of expertise and experience so as to build consensus for how best to move forward. Thankfully, several other countries are further ahead in adopting many of the recommendations outlined here, meaning there already exists a template to inform an improved data collection strategy in the United States.^92^, ^93^, ^94^, ^95^

*Deepen Understanding of the Linkages Between the Social and Subjective Indicators*. Beyond strengthening the available social and subjective indicators, research that uncovers the developing relationship between these indicators is essential. As one example, composite indices that emerge from an improved set of social indicators should be continuously validated against the expanding set of subjective indicators.^25^ More broadly, progress in this area will require a cultural shift when it comes to understanding the determinants of young people's well‐being. Rather than focusing on contextual measures that are important in the eyes of adults, researchers should be developing new tools by soliciting information directly from young people about the factors that are most important in their lives. Positioning young people as experts on their own experiences will keep measurement current, and help it adapt to new developments.

*Moving Beyond the Public Policy Mandate of Preventing Harms Towards Promoting Well‐Being*. It is challenging to offer specific policies for improving the subjective indicators of mental health and well‐being on a scientific basis, precisely because our measurement tools are not optimized for the purpose.^96^ Our best suggestion is that in addition to supporting policies promoting food security, housing affordability, unemployment support, and expansion of health care insurance and access, all of which have demonstrated associations with improved mental health,^5^, ^8^, ^97^, ^98^ policymakers begin broadening their focus beyond avoiding negative outcomes by pursuing actions that specifically enhance the positive life experiences of young people. Several evidence‐based examples include further investing in mental health promotion interventions during the school day and in after‐school settings,^99^, ^100^ improving access to arts education and participation in sports,^101^, ^102^ and enhancing neighborhood walkability and green space to promote various forms of physical activity.^103^, ^104^, ^105^ By demonstrating that policy can play a positive role in young peoples’ lives, governments will be better‐positioned to fully take advantage of the findings from the additional research outlined in the other recommendations above.

## Conclusion

The observation that children and adolescents are physically healthier than adults overlooks recent worrisome trends in their mental health and well‐being. Increases in mental distress and lower levels of happiness and life satisfaction among adolescents not only indicate that their well‐being is diminished in the present. The scale and magnitude of these shifts may actually be presaging future danger to the health of American adults, and needlessly so. Although the traditional social indicators of child and adolescent circumstances and conditions suggest that young people are thriving as never before, this conclusion is based on an incomplete model of what drives their thriving. While the improvements in these social indicators should be celebrated as important progress in certain aspects of young peoples’ lives, the divergence with the subjective indicators of mental health and well‐being means that policymakers and researchers require an updated toolkit as they search for ways to bolster young people's thriving. In brief, as the world changes, so must public‐health monitoring. Developing an integrated and holistic health monitoring system that is more up‐to‐date—responsive and sensitive to the current challenges in young peoples’ lives—is a necessary first step towards envisioning a more effective role for public health in delivering health equity for all.

*Funding Information*: This study was funded by the US Department of Health and Human Services, Agency for Healthcare Research and Quality, (Grant T32HS000046); US Department of Health and Human Services, National Institutes of Health, National Center for Advancing Translational Sciences (Grant TL1TR001883). The funder had no role in the conduct of the research nor the preparation of the article.

*Conflict of Interest Disclosures*: None declared.
