# Supplementary material for: Mixed Signals in Child and Adolescent Mental Health and Well‐Being Indicators in the United States: A Call for Improvements to Population Health Monitoring
Source: Milbank Q. 2023 Apr 13;101(2):259–86. doi: 10.1111/1468-0009.12634 (PMC10262392; doi:10.1111/1468-0009.12634)
Supplement: Supplementary file 1 [file MILQ-101-259-s001.docx]

**Appendix Material**

*_________________________ Page Number*

Appendix Figure 1: Trends in Outcome Distribution of Poor Mental Health Days in Past Month, Ages 18-25 1

Appendix A: Age-Period-Cohort Analysis Methodology 2

Appendix Table A.1: Model Selection for Combination of Age, Period, and/or Cohort Effects 6

Appendix Figure 2: Age-Period-Cohort Effects: Poor Mental Health Days, Ages 18-25; Males 7

Appendix Figure 3: Age-Period-Cohort Effects: Poor Mental Health Days, Ages 18-25; Females 8

Appendix Figure 4: Age-Period-Cohort Effects: Poor Mental Health Days, Ages 18-25; Some College or More 9

Appendix Figure 5: Age-Period-Cohort Effects: Poor Mental Health Days, Ages 18-25; High School Graduate or Less 10

Appendix Figure 6: Age-Period-Cohort Effects: Poor Mental Health Days, Ages 18-25; Annual Household Income $35,000 or More 11

Appendix Figure 7: Age-Period-Cohort Effects: Poor Mental Health Days, Ages 18-25; Annual Household Income Less Than $35,000 12

Appendix Figure 8: Age-Period-Cohort Effects: Poor Mental Health Days, Ages 18-25; Annual Household Income Less Than $35,000 13

Appendix References 14

Appendix A: Age-Period-Cohort Analysis Methodology

APC methods estimate age, period, and cohort effects within the trends of an outcome measure using a linear regression framework. ^1^ Simply regressing the outcome against all three is not possible, due to multicollinearity: cohort is an additive function of year and age. APC models make additional assumptions about the relationship of age, period, cohort in order to solve this problem of identification – a commonly used technique before more complex methods were available was to assume first two age, period, or cohort effects were identical, which is known as the Constrained Generalized Linear Model (CGLIM). However, the coefficients of the estimates in this specification have been shown to be sensitive to whichever constraint is selected. ^2^

We opt for an intrinsic estimator without any additional covariates. The intrinsic estimator is a special form of principal components regression that solves the identification problem above without requiring an arbitrary assumption that two or more of the effects are identical. ^3^ The intrinsic estimator has several desirable statistical properties, ^2^ and is commonly used in APC analysis of repeated cross-sectional data. ^4,5^ The results are interpreted as the difference in poor mental health days for any particular age, period, or cohort relative to the mean for all age-period-cohort groups combined.

We apply the population weights provided with the survey and cluster for primary sampling unit. Incorporating the two-stage sampling design with stratification is not possible given software limitations – this means the standard errors of our estimates are larger than they would otherwise be if the two-stage sampling could be fully accommodated.

For the YRBS, we group age in two year bins, both to increase sample size and because the survey is fielded biannually. We use the following groups: 14-15, 16-17, and 18+, which we label “18-19”. This drops a small portion of the high school sample that is 12-13. For the BRFSS, we have adequate sample size to separate every single year / age group / cohort.

All analyses are performed in STATA version 17. We use the *apc_ie* downloadable command for the intrinsic estimator analysis. For dichotomous outcomes, we use a logit link and convert estimates to percentages using the baseline prevalence among the entire population.

We should note that there the use of the intrinsic estimator within the field of APC models is subject to vigorous debate within the fields of sociology, demography, and epidemiology. ^6^ Our decision to use it over several alternatives is explained below:

- As previously mentioned, CGLIM models impose arbitrarily restraints about the equality of certain parameters (i.e. period 1 effect = period 2 effect). Without strong theory to select a specification, the imposition is somewhat arbitrary. Additionally, they require a reference category for each of age, period, and cohort, meaning standard errors are not estimated. Lastly, coefficient estimates that are further from the reference age, period, and cohort have widening standard errors, which are caused by the age-period-cohort matrix specification and choice of reference categories. ^2^
- Hierarchical Age Period Cohort Models have also been proposed as a method for the APC problem, and have been used in previous studies of adolescent psychological distress. ^7^ These models implement a mixed effects framework, where age and age-squared are modeled as fixed effects and cohort and period are modeled as random effects. However, these models tend to estimate smaller cohort effects relative to period effects. ^8^

Several other methodologies exist, ^6,9^ but these appear to be the main ones implemented in the literature.

Critics of the intrinsic estimator note that the results may be biased when the dimensions of the design matrix (number of age, periods, and cohorts examined) are small, particularly if there is no actual age, period, and/or cohort effect. ^10-13^ These criticisms of the methodology suggest that our estimates should be interpreted with some degree of caution. We urge fellow researchers interested in the method to consult the extensive literature on the strengths and limitations of the various models, as well as to follow Yang and Land’s prerequisites before adopting the intrinsic estimator: ^1^ 1) assessing graphical evidence of independent effects, and 2) performing a model selection analysis to compare an APC specification to those using only using a subset of the three effects see Appendix Table A.1.

Appendix Table A.1: Model Selection for Combination of Age, Period, and/or Cohort Effects

| Controls | **Adolescence**  **Outcome:** Sad or Hopeless for Two Weeks in the Past 12 Months  **Data Source:** Youth Risk Behavior Surveillance System (YRBSS)  **Ages:** 13-14,15-16,17+  **Years:** 1999-2019 (every other year) | **Young Adults**  **Outcome:** Number of Days in Poor Mental Health in Past Month  **Data Source:** Behavior Risk Factor Surveillance System (BRFSS)  **Ages:** 18-25 (every year)  **Years:** 1994-2019 (every year) |
| --- | --- | --- |
| Age | AIC: 1.2657  BIC: -1,867,462 | AIC: 19906.3  BIC: 47,560,846,927.5 |
| Period | AIC: 1.2629  BIC: -1,867,460 | AIC: 19895.6  BIC: 47,222,260,689.5 |
| Cohort | AIC: 1.2640  BIC: -1,867,400 | AIC: 19898.1  BIC: 47,302,662,297.5 |
| Age-Period | AIC: 1.2626  BIC: -1,867,447 | AIC: 19895.0  BIC: 47,203,851,525.0 |
| Age-Cohort | AIC: 1.2634  BIC: -1,867,399 | AIC: 19896.1  BIC: 47,238,025,256.2 |
| Period-Cohort | AIC: 1.2627  BIC: -1,867,330 | AIC: 19894.8  BIC: 47,197,428,000.0 |
| Age-Period-Cohort | AIC: 1.2625  BIC: -1,867,322 | AIC: 19894.9  BIC: 47,179,152,394.3 |

Notes: AIC = Akaike Information Criterion ; BIC = Bayesian Information Criterion. Lower model selection criterion generally represents a better-fitting model. In both instances, we select the full age-period-cohort model for analysis, due to its better performance in model selection, as well as simpler interpretability compared to a period-cohort model for young adults.

Appendix References

1. Yang Y, Land KC. *Age-period-cohort analysis: New models, methods, and empirical applications.* Taylor & Francis; 2013.

2. Yang Y, Fu WJ, Land KC. 2. A Methodological Comparison of Age-Period-Cohort Models: The Intrinsic Estimator and Conventional Generalized Linear Models. *Sociological Methodology.* 2004;34(1):75-110.

3. Fu WJ. Ridge Estimator in Singular Design with Application to Age-Period-Cohort Analysis of Disease Rates. *Communications in statistics-Theory and Methods.* 2000;29(2):263-278.

4. Keyes KM, Miech R. Age, period, and cohort effects in heavy episodic drinking in the US from 1985 to 2009. *Drug and alcohol dependence.* 2013;132(1-2):140-148.

5. Keyes KM, Nicholson R, Kinley J, et al. Age, Period, and Cohort Effects in Psychological Distress in the United States and Canada. *American Journal of Epidemiology.* 2014;179(10):1216-1227.

6. Fosse E, Winship C. Analyzing age-period-cohort data: A review and critique. *Annual Review of Sociology.* 2019;45:467-492.

7. Keyes KM, Gary D, O'Malley PM, Hamilton A, Schulenberg J. Recent increases in depressive symptoms among US adolescents: trends from 1991 to 2018. *Soc Psychiatry Psychiatr Epidemiol.* 2019;54(8):987-996.

8. Bell A, Jones K. The hierarchical age-period-cohort model: Why does it find the results that it finds? *Qual Quant.* 2018;52(2):783-799.

9. Fosse E, Winship C. Bounding Analyses of Age-Period-Cohort Effects. *Demography.* 2019;56(5):1975-2004.

10. Luo L. Assessing Validity and Application Scope of the Intrinsic Estimator Approach to the Age-Period-Cohort Problem. *Demography.* 2013;50(6):1945-1967.

11. Luo L. Paradigm Shift in Age-Period-Cohort Analysis: A Response to Yang and Land, O’Brien, Held and Riebler, and Fienberg. *Demography.* 2013;50(6):1985-1988.

12. Luo L, Hodges J, Winship C, Powers D. The sensitivity of the intrinsic estimator to coding schemes: Comment on Yang, Schulhofer-Wohl, Fu, and Land. *American Journal of Sociology.* 2016;122(3):930-961.

13. O’Brien RM. Constrained Estimators and Age-Period-Cohort Models. *Sociological Methods & Research.* 2011;40(3):419-452.
